# Complication neurologique au cours de la polyarthrite rhumatoïde: tétraparesie par luxation C1-C2

**DOI:** 10.11604/pamj.2015.21.199.7255

**Published:** 2015-07-15

**Authors:** Mouna Sghir, Sonia Jemni

**Affiliations:** 1 Unité de Médecine Physique, CHU Taher Sfar, Mahdia, Tunisie; 2 Service de Médecine Physique et Réadaptation Fonctionnelle, CHU Sahloul, Sousse, Tunisie

**Keywords:** Polyarthrite rhumatoide, subluxationc1-c2, tétraparésie, compression médullaire, Rheumatoid arthritis, C1-C2 subluxation, tetraparesia, spinal cord compression

## Image en medicine

Les lésions de l'articulation atloïdo-axoïdienne peuvent émailler l’évolution de diverses pathologies rhumatismales. Elles peuvent se compliquer d'un handicap fonctionnel important et d'une atteinte neurologique parfois sévère. Nous rapportons le cas d'une patiente âgée de 55 ans, diabétique, hypertendue et suivie en Rhumatologie depuis 15 ans pour une polyarthrite rhumatoïde séropositive destructrice et déformante. Elle a été mise initialement sous MTX et SLZ, mais la polyarthrite rhumatoïde est restée évolutive d'où sa mise sous Actemra. Par ailleurs, un bilan radiologique standard demandé pour des cervicalgies révèle un diastasis C1-C2. Une TDM cervicale a montré une atteinte avancée et diffuse du rachis cervical avec une luxation C1-C2, mixte, antérieure, rotatoire et verticale et une subluxation C4-C5 et l'IRM cervicale a montré une compression médullaire pour laquelle l'abstention chirurgicale est décidée et la patiente est mise sous collier cervical. Quelques mois après, elle se présente pour une lourdeur des deux membres inférieurs avec des troubles de la marche d'installation progressive et s’étendant aux membres supérieurs. L'examen neurologique trouve une tétraparésie prédominante à gauche, un syndrome quadripyramidal et un syndrome cordonal postérieur. Ainsi, la patiente a été opérée: laminectomie C1 et ostéosynthèse. En post opératoire, lle a bénéficié de séances de rééducation fonctionnelle d'un appareillage à type d'orthèses de repos pour les deux mains et d'une attelle tibio-tarsienne gauche.

**Figure 1:**
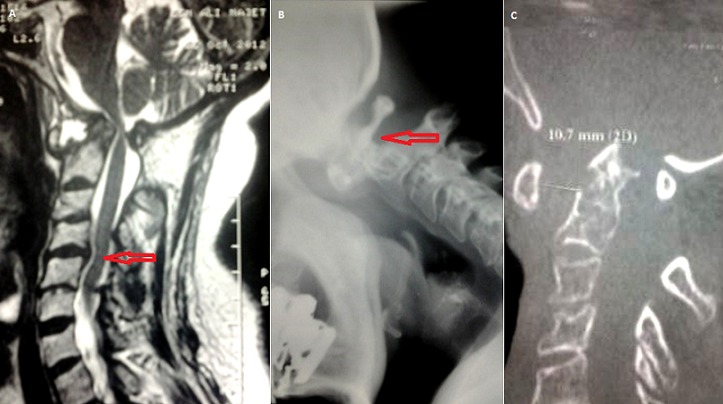
(A) IRM cervicale (coupe sagittale en T2): compression médullaire; (B) Radiographie du rachis cervical en flexion: diastasis C1-C2; (C) TDM cervicale: luxation C1-C2 et une subluxation C4-C5

